# Consistency of Bacterial Triggers in the Pathogenesis of Hidradenitis Suppurativa

**DOI:** 10.3390/vaccines11010179

**Published:** 2023-01-13

**Authors:** Elia Rosi, Prisca Guerra, Gianmarco Silvi, Giulia Nunziati, Ilaria Scandagli, Antonella Di Cesare, Francesca Prignano

**Affiliations:** Department of Health Sciences, Section of Dermatology, University of Florence, 50125 Florence, Italy

**Keywords:** hidradenitis suppurativa, acne inversa, skin microbiome, gut microbiome, microbiota, bacteria, microbiology, microorganisms, biofilm, pathogenesis

## Abstract

Hidradenitis suppurativa (HS) is an inflammatory skin disease whose pathogenesis remains poorly defined. Over the past decades, the bacterial role in HS patients has been a focus of research. According to the literature, the HS skin (and probably gut) bacterial composition is different to that of healthy controls. To date, a key question is whether compositional changes in the microbial populations are responsible for the development of HS (primum movens), or only secondarily reflect the ongoing inflammatory process. The great diversity of methodologies that have been used to study microbial role in HS have led to an accumulation of conflicting results. Thus, in view of these considerations, the aim of this article is to provide the reader with an overview about different hypotheses proposed to explain the bacterial role in HS pathogenesis.

## 1. Introduction

Hidradenitis suppurativa (HS), also known as acne inversa or Verneuil’s disease, is a debilitating, chronic, inflammatory skin disease. The pathogenesis of the disease still remains not fully elucidated [1]. Although clinical presentation of HS is reminiscent of bacterial infection, there is no evidence to consider bacteria as infectious pathogens according to Koch’s postulates [2]. The aberrant innate immune response might influence bacterial dysbiosis, generating chronic inflammation [3,4,5]. The relationship between bacterial microorganisms and HS has been the focus of attention of the scientific world in recent years. Indeed, findings from studies investigating microbial composition of both lesional and unaffected HS skin helped researchers to better understand the role of bacteria in HS pathogenesis. In recent years, metagenomics studies have further improved our understanding of microbial population characterization in HS patients [6]. Overall, both culture and metagenomic studies described an increased abundance of anaerobes and opportunistic pathogens with a decrease in normal skin commensals (such as *Cutibacterium*), thus supporting a dysbiotic skin microbiome in HS patients [7,8,9,10,11]. In addition, the role of the gut microbiome in HS is a new emerging field of research [12]. Nevertheless, the biological significance of bacterial microorganisms in HS pathogenesis has not yet been defined. A key question is whether the observed cutaneous dysbiosis (i) initiate inflammation in HS (primum movens), (ii) is the adaptive response to acute inflammation, or iii) actively take part in HS pathophysiology during disease progression [13].

Thus, in view of these considerations, the aim of this article is to provide the reader with an overview about different hypotheses proposed to explain the bacterial role in HS pathogenesis.

## 2. Methods

A literature search in the electronic database PubMed was conducted up to the 31st of October 2022, using the search terms “hidradenitis suppurativa” OR “acne inversa” in combination with “bacteria” OR “microbiome’” OR “microorganisms” OR “bacteriology” OR “microbiology”, to identify relevant English-language publications. All articles were screened based on the content of the title and abstract to select the studies that assessed the cutaneous and/or gut bacterial flora in HS, formulating a hypothesis about its pathogenetic role in the disease. References of relevant articles were also manually searched for possible inclusion in the present review. As a result of the methodological criteria applied in this review, there was a chance to exclude relevant articles based on not satisfying any of these criteria.

## 3. Microbes and HS: Evidence to Date

To the best of our knowledge, the present review is based upon the available studies focusing on bacteria in HS to date (Table 1).

Highet and colleagues were among the first researchers to hypothesize that secondary bacterial infections could be responsible for HS flare-ups. Their findings suggested a role for *S. milleri* as the main microbial pathogen in perineal actively discharging lesions. Indeed, the presence and the disappearance of this bacterium were significantly associated with disease activity and clinical improvement, respectively [14].

Eight years later, in 1996, Jemec at al. found microorganisms in only approximately 50% of examined aspirates. The significantly shorter disease duration for patients in whom *S. aureus* was found, led the authors to hypothesize its temporary role in the early phase of HS pathogenesis, e.g., by changing the anatomical structure of the hair follicles [15].

Additional data on *S. aureus* nasal colonisation in HS patients was reported by three studies. Katoulis and colleagues first, in their observational cohort study, observed 22.1% prevalence of nasal carriage status of *S. aureus* among 68 HS patients, thus concluding that eradicating this colonization might decrease disease activity [16]. Dinh et al., in their cross-sectional study, estimated a lower prevalence of *S. aureus* nasal colonisation among HS participants in comparison with a control group in a large cohort of Danish blood donors, not supporting the hypothesis of nasal colonisation as a driver of HS [17]. Recently, Stergianou and colleagues, in their retrospective observational study, proposed that *S. aureus* nasal carriage status might have a role as a driver of HS flare-ups in adalimumab-treated patients [18]. Adalimumab improves acute inflammation but would not recover cutaneous dysbiosis (and permanent skin damage) in severe HS patients, as demonstrated by the not significant temporal evolution of the skin microbiome during adalimumab treatment [19]. According to Giamarellos-Bourboulis, the hypothesis for a role of *S. aureus* skin colonization in HS flare-ups is supported by (i) the attenuated ability of phagocytosis of *S. aureus* by macrophages (in which the phagocytic capacity is inhibited by overproduction of interleukin (IL)-26 in HS); (ii) the greater production of human β-defensin–2 (hBD-2) by whole blood stimulated with heat-killed *S. aureus* in HS patients compared with healthy control subjects [53]. A recent study analysed whether Panton-Valentine leucocidin (PVL), a known virulence factor of *S. aureus*, might negatively influence the HS clinical course. Interestingly, among the 5 *S. aureus* strains isolated, none of them were positive for PVL, in contrast to the control group, where 26.3% tested positive for PVL. The study results seemingly point against a pathogenic involvement of *S. aureus* producing PVL in HS [20].

The decrease in tryptophan (Trp) levels in HS skin lesions might confer a selective (growth) advantage to *S. aureus* (a Trp-independent pathobionts) [21].

The polymicrobial (aerobic and anaerobic) nature of axillary HS (2.5 isolates per specimen) reported by Brook et al. in their retrospective study might suggest that the synergism of bacterial species has a pathogenic role in the development of HS lesions [22]. In 1998, Lapins and colleagues detected bacterial growth in deeper levels of samples from HS lesional skin, thus excluding (with a laser CO2 surgical method) contamination from superficial commensals bacteria. They isolated coagulase-negative staphylococci (CNS) and *S. aureus* in 84% and 56% of HS patients, respectively. Furthermore, the researchers observed, at deeper levels, aerobic and anaerobic bacteria in 60% and 81% of cultures, respectively. Thus, they supposed that sterile inflammatory lesions are exceptional in HS and suggested that aerobic and anaerobic bacterial colonization might follow the rupture of the pilosebaceous unit. Moreover, the authors hypothesized that hair fragments and corneocyte debris from the hair follicle rupture might act as a foreign body, in a context of the inflammatory background, enhancing the pathogenic properties of CNS [23]. Subsequently, Sartorius et al., on the one hand, confirmed the possible pathogenetic role of CNS in HS exacerbations, on the other hand, they did not detect *S. aureus* in any acute HS lesions, thus hypothesizing that CNS might contribute more than *S. aureus* in inducing a non-infectious inflammatory process in HS [24]. Matusiak’s working group added evidence to the presence of a polymicrobial flora in HS patients. In their study, all but one of the HS subjects had a positive culture, with up to five different bacterial species. The polymicrobial flora consisted primarily of three groups: staphylococci (CNS were the most commonly isolated organisms, followed by *S. aureus*, mainly in patients with shorter disease duration), bacteria of intestinal flora (Enterococci and Enterobacteriaceae) and anaerobes [25]. Conversely, in a 2015 study, Katoulis and colleagues found fewer HS patients with positive cultures (68%) for a variety of aerobic and anaerobic bacteria, but only one among them had a polymicrobial flora. Therefore, the authors were hesitant to exclude bacterial contamination as a secondary phenomenon in HS lesions [26]. The identification of two bacterial pathogens species associated with HS lesions, *S. lugdunensis* (a skin commensal of the lower extremities and inguino-perineal area) and anaerobic actinomycetes (common inhabitants of the mouth and gastrointestinal tract), led Guet-Revillet and colleagues to hypothesize their role in HS pathogenesis, especially in nodules and abscesses, as well as in chronic deep-seated lesions, respectively [27].

In 2019, Ardon et al. analysed the in vitro characteristics of *S. epidermidis* isolated from HS patients. They observed an increased virulence in *S. epidermidis*, characterized by (i) resistance to commonly used antibiotics in HS, (ii) greater biofilm production and (iii) more difficult biofilm eradication. Nevertheless, not all *S. epidermidis* strains isolated from lesional skin demonstrated biofilm formation ability [28]. In a later study, Ardon and colleagues observed biofilm producing capacities and an increased resistance to antibiotics in *S. lugdunensis* strains of HS patients compared to the healthy control’s strains. The authors hypothesized that these bacterial characteristics might promote disease activity. Interestingly, they observed that biofilm eradication with clindamycin occurred in all strains of *S. lugdunensis*, even in one resistant to the antibiotic, suggesting that clindamycin’s biofilm degradation ability is independent from its bactericidal activity [29].

In a cross-sectional study on 50 HS patients, Nikolakis et al. evaluated the relation between bacterial species and disease severity. The authors observed that a higher Hurley stage correlated with the presence of a more polymicrobial flora. The co-isolation of both aerobic and anaerobic species was a significant independent predictor for greater disease severity in both univariate and multivariate models. Moreover, they demonstrated that certain species were associated with a higher Hurley score, in particular *S. aureus*, Streptococci, Enterobacteriaceae and obligate anaerobic Gram-negative rods. Notably, the presence of staphylococci in Hurley stages I and II was rather minimal as opposed to Hurley stage III patients, in accordance with previous studies, suggesting that *S. aureus* is involved in later disease stages. These observations led them to hypothesize that bacteria might indeed worsen HS, enhancing the innate immune reaction. They deemed the hypothesis of bacterial colonization as a neutral bystander less likely, considering the improvement of inflammation induced by antibiotic treatments [30]. In a recent work, Benzecry and colleagues observed positive cultures in approximately 50% of samples from draining lesions of HS patients. Interestingly, the culture-positive rate increased significantly with greater disease severity. In particular, 0%, 28% and 67% of cultures were positive in stage I, II and III, respectively. The more prevalent isolates were *Proteus mirabilis* and *S. aureus*. The authors agreed on the widely discussed hypothesis of a secondary involvement of bacteria in the pathogenesis of HS, particularly in established lesions, as a maintaining factor for chronic suppurative lesions. However, they were not able to establish whether and which specific group of bacteria played a role in this disease [31]. Rather recently, Naik at al., observing a relative abundance of mixed anaerobes and a relative decrease in the major commensal bacteria at both unaffected and affected HS body sites, confirmed that the skin microbiome differs between HS patients and healthy volunteers. Furthermore, they found, on the one hand, a positive association between HS severity and this relative abundance of anaerobes and, on the other hand, a negative correlation with major skin commensals. The researchers also hypothesized that gut and vaginal anaerobic bacteria might colonize inguinal folds because of the proximity of anatomical sites [32]. Conversely, an analysis of the cutaneous mycobiome using high throughput sequencing (HTS) demonstrated comparable follicular mycobiomes in HS patients (both in lesional and non-lesional skin) compared with healthy controls, thus ruling out a central role of the mycobiome in HS pathogenesis [54].

Investigating the skin microbiome in clinically unaffected skinfolds (through both conventional culturing and 16S rRNA amplicon sequencing), Riverain-Gillet et al. found (i) a decreased abundance of CNS, particularly of *S. epidermidis* and (ii) a higher prevalence of anaerobes in HS skinfolds compared to controls, particularly of *Prevotella*. Moreover, the researchers observed that increased body mass index (BMI) and gluteal localization contributed to shifting the skin microbiome into an increasingly anaerobic one. Notably, the dysbiosis observed was reminiscent to that of HS lesions. Nevertheless, the authors were not able to assess whether this dysbiosis was (i) a result of the specific growth conditions in occluded follicles, (ii) a trigger or (iii) a result of HS chronic inflammation [33].

Hessam and colleagues in their retrospective study analysed bacterial cultures obtained from deep portions of HS lesions. The study provided evidence for a significantly greater frequency of CNS, *E. coli*, and polymicrobial flora in cultures obtained from the axillae, the groin, and the gluteal/perineal area, respectively. The researchers concluded that microbial flora in HS lesions reflects skin commensal flora. They considered commensal skin bacteria colonization of HS lesions as a secondary event that follows the occlusion of the follicular ducts and the consequent rupture of the hair follicle. The spill of bacteria into the dermis recruits an increasing number of immune cells. Indeed, through the recognition of bacterial lipopolysaccharide (LPS), toll-like receptors (TLRs) may induce the production and release of cytokines and chemokines by keratinocytes and macrophages. Thus, bacteria may play a role in initiating and maintaining the self-perpetuating vicious circle of inflammation [34]. Even though Jamalpour et al. found a little bit different topographical distribution of bacterial species (*S. aureus* and *Diphtheroid* were more frequent in cultures obtained from the axilla and *E. coli* was more frequent in axilla and groin), they support the pathogenetic mechanism reported by their German colleagues [35].

Guet-Revillet and colleagues confirmed that skin dysbiosis may have a role in HS pathogenesis. In their prospective study, they found a predominance in lesions of two Gram negative anaerobic rod taxa, *Prevotella* and *Porphyromonas* (commensal bacteria of the oropharyngeal and vaginal tracts). The perturbations in the healthy microbiome may lead to selection and expansion of these opportunistic microbes, which in turn revert to their pathogenic phenotype. Thus, the authors hypothesized that HS is firstly a skin barrier disease, predisposing patients to recurrent infections. In addition, the dysregulated production of antimicrobial peptides, as well as the pro-inflammatory phenotype of keratinocytes, may favour the local persistence of these pathobionts [36].

In 2019, Ring et al. analysed, through next-generation sequencing (NGS), the bacterial composition of HS sinus tracts (which differs from that of Crohn’s disease fistulas [55]), primarily detecting anaerobic species such as *Porphyromonas* spp. and *Prevotella* spp. Notably, these two genera were only found through NGS studies, and not by using conventional culturing methods. This finding suggests that the bacterial microbiome in enclosed HS cavities is mainly composed by non-cultivable bacteria. Moreover, the authors detected a scarce presence of *Propionibacterium* spp., normally dominating the intertriginous microbiota. The presence of *Porphyromonas* spp. and *Prevotella* spp., usually present in the mucocutaneous surface microbiota, and not in the intertriginous one, and the efficacy of antibiotics covering these anaerobic species, led the researchers to suggest their pathogenetic involvement in HS, either as a driver or biomarkers [37].

Ring et al., in their NGS case-control study, found an abundance of *Corynebacterium*, *Porphyromonas*, and *Peptoniphilus* species in the HS lesional skin, whilst *Acinetobacter* and *Moraxella* species dominated the non-lesional skin. Furthermore, they reported a significant reduced presence of *Propionibacterium* spp. in HS skin compared with normal skin [38]. Similar findings were reported in 2020 by Schneider et al. in their cohort of 10 normal subjects and 11 HS subjects. Indeed, the researchers found a reduced presence of *Cutibacterium* (formerly known as *Propionibacterium*) and an abundance of *Peptoniphilus* and *Porphyromonas* in HS skin. The authors also analysed the functional impacts of the dysbiotic communities, applying a computational approach [39]. A predictive metagenomics analysis was also performed by Ring and colleagues on their previously published data. Their data identified significantly different pathways between the lesional samples and healthy controls (DNA replication, cell cycle, mismatch repair, and the biosynthesis of peptidoglycan and ansamycins, highly associated with lesional samples; the metabolism of several amino acids was inversely associated with the lesional samples) [56]. Comparisons between Schneider et al. (2020) and Ring et al. (2017) data sets using two different pipelines was performed by Schneider and colleagues. In their predictive metagenomic analyses, the researchers identified a total of nine pathways in common between both data sets and analyses pipelines. The authors confirmed a possible higher microbial turnover and/or proliferation in HS skin, whereas glyoxylate and dicarboxylate metabolism was significantly decreased in HS lesional skin [57].

The reduced number and volume of sebaceous glands (and consequently of sebum production) in HS skin might lead to cutaneous dysbiosis, favouring the lack of commensal *Cutibacterium* species, whose antimicrobial activity against opportunistic pathogens (such as *Peptoniphilus* spp. and *Porphyromonas* spp.) would thus decrease [38,39,56,57]. It is therefore reasonable to propose probiotics in HS as a potential treatment option [58].

Interestingly, Antal and colleagues compared the skin flora of three distinct skin niches (lesional moist, and unaffected dry and sebaceous skin regions) of HS patients and the corresponding regions of healthy controls. The study showed (i) a reduced total number of detected species from the distinct skin sites of HS patients compared with controls, (ii) a uniform reduction of *Lactobacillus* spp. in HS skin compared to healthy controls and (iii) a high abundance of Gram-positive anaerobic cocci in HS lesions compared with the corresponding moist regions of controls. Thus, the researchers hypothesised that the reduction of both species diversity and *Lactobacillus* spp. (with immune-modulatory capacity) in HS patients might favour anaerobe species colonization at lesional sites [40].

The decrease in both the abundance of *Lactobacillus* spp. (Trp-metabolizing strains) and Trp availability in HS skin lesions might impair the production of the bacteria-derived aryl hydrocarbon receptor (AHR) agonist indole-3-acetic acid (IAA, Trp metabolite), thus resulting in a defective activation of AHR (whose role is critical for the regulation of skin inflammation) [21].

Analysing bacterial growths in HS patients, Bettoli et al. observed the resistance to clindamycin, rifampicin and tetracyclines in 65.6%, 69.3% and 84.5% of bacterial isolates, respectively. The most frequently isolated species were *Proteus* spp. (13.5%), *E. Coli* (9.8%), *S. epidermidis* (9.2%) and *S. Agalactiae* (8.6%), supporting the hypothesis of commensal skin bacteria invasion as a secondary event in HS lesions. Thus, the researchers hypothesized that the beneficial effect of tetracycline and of the association of rifampicin and clindamycin, is more probably ascribable to their immunomodulatory effect. Alternatively, it might be justified by an anti-microbial effect directed towards anaerobes’ bacteria, demonstrated to be predominant in HS lesions in the literature, that were probably not isolated in this study due to the limited duration of culture incubation [41].

Notably, some anaerobic microorganisms recognized as part of the HS microbiota may generate dysbiosis and superinfection, as reported in two cases from the literature: a case of multiple abscess by *Actinotignum schaalii* and *Prevotella melaninogenica* in the perineal area of an HS patient [59], and a case of severe HS associated with actinomycosis by *Actinomyces meyeri* [60].

### 3.1. Biofilm

The role of biofilm in HS pathogenesis is widely discussed in the existing literature. In 2012, Kathju et al. for the first time hypothesised that HS might be considered a biofilm disease. In accordance with the criteria proposed by Parsek and Singh (2003), they observed bacteria in clusters adherent to the luminal surface of the sinus tissue of an HS patient. According to the authors, the presence of biofilm might explain HS resistance to antibiotic therapy and recurrences after treatment. Finally, to explain efficacy of the anti-tumor necrosis factor (TNF)-alpha, the researchers theorized that tissue damage is not due to biofilm itself, but by its ability to foster the inflammatory response [61]. Jahns and colleagues, in their retrospective research, found bacteria in 50% of the hair follicle and sinus tracts of HS patients. In contrast to previous findings, the study group detected neither *S. aureus* nor CNS in any sample. Indeed, the observed bacteria were located predominantly in anaerobic sites of hair follicles and sinus tracts, whereas *Staphylococcus* spp. are of an aerophilic nature. Furthermore, in 20% of HS patients, biofilm like-structures were observed [42]. In 2016, Okoye et al. found evidence of biofilm presence in only 2 out of 10 samples from acute HS lesions and in none of the non-affected skin samples. Thus, they suggested that biofilm might play a role in the late pathogenesis of HS rather than in its initiation. The researchers further hypothesized that the absence of biofilm in acute lesions might be an indicator of the presence of *S. epidermidis* in early HS [43]. Indeed, in a previous study, Iwase and colleagues demonstrated that biofilm formation might be inhibited by the presence of skin commensal such as *S. epidermidis* [62]. In a 2017 observational prospective histological study, Ring et al. found the presence of biofilm in 67% and 75% of lesional samples and perilesional samples, respectively. Interestingly, bacterial aggregates >50 μm in diameter were observed in regions such as the infundibulum (39%) and sinus tracts (50%). They hypothesized that the typical HS lesions, such as deep-seated nodules, dilated hair follicles and sinus tracts, might foster bacterial biofilm growth for their anoxic environment. Scattered intradermal corneocytes and hair fragments, acting as foreign bodies, contribute, as a nidus, to the formation of biofilms by commensal bacteria. Furthermore, the authors suggested that biofilm aggregates, with their immunological footprint, might lead to the pro-inflammatory cytokine mix found in HS lesions. Finally, the researchers hypothesised that biofilm might be responsible for the resistance towards antibiotics of HS lesions, and the improvement of the disease shown with combined medical and surgical therapy might reflect the removal of biofilm [44]. Subsequently, Ring and colleagues conducted a case-control study to assess whether HS patients’ healthy skin was also characterized by biofilm presence. Surprisingly, they detected fewer bacteria and less biofilm in clinically non-affected axillary skin of patients with HS compared with healthy controls. They therefore hypothesized that the altered microbiota found in clinically non-affected HS skin might lead to greater susceptibility to pathogens’ invasions or even trigger immuno-mediated inflammation. The authors further suggested that the presence of biofilm found in lesional HS skin might reflect a secondary colonization of biofilm-producing bacteria, fostered by the rupture of the innate skin barrier [45]. Further investigating this aspect, Kjærsgaard Andersen et al. analysed the inflammatory infiltrate, searching for the expression of CD4, CD8, CD25, FoxP3 and IL-17 in clinically uninvolved follicles of HS patients versus healthy controls. Firstly, they observed the presence of bacterial biofilm in only 12.5% of HS patients compared to 85% of healthy controls, confirming the previous finding. Secondly, HS correlated with a significantly higher number of CD4+ and CD4+FoxP3+ and CD25+ cells, but not of CD8+, CD25FoxP3+ cells or IL-17. In particular, HS patients with biofilm had significantly greater numbers of CD4+ FoxP3+ cells compared to the other groups. These results led the researchers to hypothesize that biofilm in HS patients might heighten the expression of regulatory T- cells and thus their immunomodulatory capability. Therefore, whereas a normal symbiont biofilm on the skin might be protective, the lack or an altered epidermal biofilm in pre-clinical HS might lead to a higher vulnerability to invading pathogens, or, alternatively, might have a disadvantageous immune modulating effect [46].

### 3.2. The Peripheral Blood Bacterial Compostion

Researchers working on the composition of bacteria in the blood of HS patients started by assuming that the symptomatology in HS patients could result from a low-grade bacteraemia. Nevertheless, Ring et al. did not find an altered systemic bacterial composition compared to that of healthy controls [47]. Conversely, Hispán and colleagues reported that the presence of bacterial DNA in the peripheral blood of HS patients was more common compared to age-and gender-matched healthy blood donors (34.0% vs. 4.0%, *p* < 0.001). Furthermore, the bacterial species most frequently identified in HS patients were Gram-negative bacilli (82.4%), especially *E. coli*. Thus, the researchers hypothesized the existence of a pathogenic circle: i) systemic chronic inflammation associated with HS increase gut barrier permeability leading to bacterial translocation from the intestinal lumen to the bloodstream; ii) the presence of bacterial products in the bloodstream promotes the release of pro-inflammatory cytokines (as demonstrated by the significant association between presence of bacterial DNA in blood and increased serum levels of TNF, IL-1β and IL-17A) perpetuating systemic low-grade inflammation [48].

### 3.3. The Gut Bacterial Composition

The recent research has focused on investigating the association between HS and gut microbial dysbiosis. Kam et al., in their pilot case series study, found that HS patients had significantly decreased gut microbiota species diversity (expressed as reduced Shannon Diversity Index) compared with healthy controls. The study showed an increase in the *Bilophila* and a reduced abundance in the *Lachnobacterium* species in HS patients. According to authors, *Bilophila* might be responsible for exacerbating the effects of high-fat diets on intestinal barrier dysfunction, whereas a decrease in the butyrate- (a fatty acid with anti-inflammatory properties) producer *Lachnobacterium*, might be involved in systemic inflammation [49]. Instead, a depletion of protective *Faecalibacterium prausnitzii* (reported in psoriasis and inflammatory bowel disease) was not observed in HS patients [50]. Interestingly, Lam et al. speculated about a possible role for *Robinsoniella peoriensis*, which was found in 59% of HS faecal samples and in none of the healthy controls, as a potential pathogen in HS [51].

Rather recently, McCarthy and colleagues compared the microbiota across skin, nasal mucosa, and faeces of HS patients with healthy controls. Interestingly, the authors found (i) more elevated levels of *Ruminococcus gnavus* in the faecal microbiome of HS individuals (also reported in Crohn’s disease); (ii) a relative higher abundance of *Finegoldia magna* in HS compared with healthy controls in skin samples. Thus, the researchers hypothesized a role for both in HS pathogenesis. Indeed, the production of TNF-alpha might be induced, via TLR4, by a polysaccharide produced by *R. gnavus* (as experimentally demonstrated in Crohn’s disease), whereas *F. magna* might stimulate the formation of neutrophil extracellular traps (NETs) [52].

According to Molnar et al., gut dysbiosis might influence the development and maintenance of HS lesions and tracts. Indeed, an increase in the *Firmicutes*/*Bacteroidetes* ratio (associated with a reduction in antimicrobial peptides), induced in turn by a high fat diet, would lead to an increase in the production of inflammatory cytokines by the intestinal epithelia and in the expression of matrix metalloproteinases (MMPs) [63].

## 4. Conclusions

HS is associated with an imbalance in the skin (probably also gut) microbiome based on data from reported studies. Several hypotheses have been proposed by researchers to explain how bacteria may contribute to the pathogenesis of the disease but, to date, it is not yet possible to understand whether skin (and gut) dysbiosis is a necessary (but not sufficient) etiological factor or epiphenomenon. Understanding the real role of microorganisms in HS remains a challenge, primarily due to the great variability of methodology (including the variability of anatomical sites and lesions assessed) across studies. Thus, the standardization of methods, reducing variability and ensuring the accuracy of skin microbiome data, will be the key to the success of the future interdisciplinary research (which will involve microbiology, molecular biology, omics studies, as well as biochemistry) [64,65]. The skin microbiome as a biomarker for dermatological drug development should also be explored [66].

Thus, in view of these considerations, future studies should focus on (i) the role of the skin (and gut) microbial flora in the early pre-clinical stages of disease pathophysiology [67] (in areas where there are no active HS lesions); (ii) the identification of biomarkers able to distinguish between superinfection and “pathobiont super-colonization”; (iii) the real role of the biofilm in HS as the “microorganism sancta sanctorum” (inaccessible to external treatments); (iv) the use of skin microbiome manipulation strategies (such as skin microbiome transplant, skin bacteriotherapy, prebiotics, probiotics and postbiotics stimulation [68]) as a valid therapeutic alternative to antibiotics for reducing resistance.

## Figures and Tables

**Table 1:** A summary of studies focusing on bacteria in HS.

Reference	Type of Study	Study Participants	Most Common Bacteria and Other Relevant Findings
[14]	Culture study	32 HS patients	*Streptococcus milleri*; *Staphylococcus aureus*; anaerobic streptococci; *Bacteroides* spp.; Coliform bacteria; *Proteus* spp.
[15]	Culture study Serological study	41 HS patients	*Staphylococcus epidermidis*; *S. aureus*; *S.* milleri; polymicrobial
[16]	Culture study	68 HS patients	*S. aureus* carriage rate (25%); (35% MRSA)
[17]	NA	5218 blood donors (117 HS patients and 5101 healthy controls)	*S. aureus* nasal carriage rate: 33.3% HS vs. 41.4% healthy controls
[18]	Culture study	39 HS patients treated with adalimumab	Carriage for *S. aureus* was detected in 5 (50%) patients who failed to achieve HiSCR at 12 weeks
[19]	Sequencing study	22 HS patients 12 healthy controls	*Prevotella* spp. and *Peptoniphilus* spp. at HS lesional sites *Paucibacter* spp. and *Caulobacter* spp. in healthy controls Not significant temporal evolution of microbiome during adalimumab treatment
[20]	Culture study PCR analysis	30 HS patients	*S. aureus*; CNS; Peptostreptococcaceae; Enterobacteriaceae
[21]	Sequencing study	8 HS patients 9 healthy controls	*Corynebacterium* spp. and anaerobic bacteria
[22]	Culture study	17 HS patients	*S. aureus*; *Streptococcus pyogenes*; *Pseudomonas aeruginosa*; *Peptostreptococcus*; *Prevotella*
[23]	Culture study	25 HS patients	*S. Aureus*; CNS; *Peptostreptococcus* spp.; *Propionibacterium acnes*
[24]	Culture study	10 HS patients	CNS; *Corynebacterium* spp.; Anaerobic Gram-positive cocci; Micrococci; *Clostridium* spp.
[25]	Culture study	28 HS patients	*S. epidermidis*; *Proteus mirabilis*; *S. aureus*; *Enterococcus faecalis; Escherichia coli*
[26]	Culture study	22 HS patients	*P. mirabilis*; *Staphylococcus haemolyticus*; *Staphylococcus lugdunensis*; *Dermacoccus nishinomiyaensis* and *Propionibacterium granulosum*
[27]	Culture study Sequencing study	82 HS patients	Gram-positive cocci; *Prevotella* spp.; *Porphyromonas* spp.; *Bacteroides* spp.; *Fusobacterium* spp.
[28]	Culture study	26 HS patients	*S. epidermidis* (89% isolates were strong biofilm producers *in vitro*)
[29]	Culture study	26 HS patients 1 healthy control	*S. lugdunensis* (100% strong biofilm producers)
[30]	Culture study	50 HS patients	Anaerobic non-Enterobacteriaceae; Enterobacteriaceae; Coagulase-positive staphylococci; CNS; Anaerobic enterococci
[31]	Culture study	46 HS patients	Enterobacteriaceae; *Streptococcus* spp.; *Corynebacterium* spp.; *Staphylococcus* spp.; Anaerobic Gram-positive and Gram-negative bacteria
[32]	Sequencing study	12 HS patients, 5 healthy controls	Increased relative abundance of Gram-negative anaerobes Increased relative abundance of Gram-positive anaerobes Decreased relative abundance of *Cutibacterium* spp.
[33]	Culture study Sequencing study	60 HS patients 17 healthy controls	Increased abundance of anaerobes Decreased abundance of skin commensals
[34]	Culture study	113 HS patients	CNS; *S. aureus*; *P. mirabilis*; *E. coli*; *Corynebacterium* spp.; *Enterococcus* spp., Viridans streptococci; polymicrobial (45.1%)
[35]	Culture study	26 HS patients	*S. aureus*; *Diphtheroid*; *E. coli*
[36]	Culture study	65 HS patients	Anaerobes; *Streptococcus anginosus*, *Actinomyces* spp.; *S. aureus*
[37]	Sequencing study	32 HS patients	5 microbiome types identified: *Porphyromonas* spp. (type I) *Corynebacterium* spp. (type II) *Staphylococcus* spp. (type III) *Prevotella* spp. (type IV) *Acinetobacter* spp. (Type V)
[38]	Sequencing study	30 HS patients 24 healthy controls	5 microbiome types identified: *Corynebacterium* species (type I) *Acinetobacter* and *Moraxella* species (type II) *S. epidermidis* (type III) *Porphyromonas* and *Peptoniphilus* species (type IV) *P. acnes* (type V)
[39]	Sequencing study	11 HS patients 10 normal subjects	Decreased relative abundance of skin commensals and increased abundance of opportunistic anaerobic pathogens
[40]	Culture study	11 HS patients 14 age- and sex-matched healthy controls	Decreased presence of *Lactobacillus* spp.; *Cutibacterium acnes* and *Staphylococcus caprae* Increased abundance of *E. faecalis*
[41]	Culture study	137 HS patients	*Proteus* spp.; *E. coli*; *S. epidermidis*; *Streptococcus agalactiae*
[42]	Immunolabelling study	27 HS patients	Untyped small coccoidal bacteria; *P. acnes;* biofilm-like structures 1/5 of HS patients
[43]	Epifluorescence microscopy	10 HS patients	Biofilms found in 2 of the acute HS lesions and not in any of the uninvolved skin samples
[44]	PNA- FISH in combination with CLSM	42 HS patients	Biofilm found in 67 % of the lesional samples and 75% of the perilesional samples Cocci-like bacteria
[45]	PNA-FISH probes in combination with CLSM	24 HS patients 24 healthy controls	12% of the HS samples were categorized as positive for small aggregates or single scattered cells Predominant morphology cocci and rod shape
[46]	Histologic material stained for CD4, CD8, CD25, FoxP3 and IL17	16 HS patients 21 healthy controls	12.5% of HS patients had bacterial biofilm in their axilla vs. 85% of the healthy controls
[47]	Culture study Sequencing study	27 HS patients 26 healthy controls	No different bacterial composition between HS patients and healthy controls (blood)
[48]	Sequencing study	50 patients with HS 50 matched controls	*E. coli*; *Klebsiella pneumoniae* and Gram-positive cocci (blood)
[49]	Sequencing study	3 HS patients 3 healthy controls	Increased abundance of *Bilophila* and *Holdemania* Decreased abuncance of *Lachnobacterium* and *Veillonella* (gut)
[50]	Sequencing study	34 HS patients (17 with concomitant IBD) 42 psoriasis patients (13 with IBD) 31 IBD patients 33 healthy controls	No depletion of *Faecalibacterium prausnitzii* (gut)
[51]	Sequencing study	17 HS patients 20 healthy controls	*Robinsoniella peoriensis* and *Sellimonas* (gut)
[52]	Sequencing study	59 HS patients 30 healthy controls (fecal samples) 20 healthy controls (nasal and skin swabs)	*Ruminococcus gnavus* and *Clostridium ramosum* (gut)

Abbreviations: HS, hidradenitis suppurativa; MRSA, meticillin resistant Staphylococcus aureus; HiSCR, The Hidradenitis Suppurativa Clinical Response; PCR, polymerase chain reaction; CNS, Coagulase-Negative Staphylococci; PNA, Peptide Nucleic Acid; FISH, Fluorescence in situ Hybridization; CLSM, Confocal Laser Scanning Microscopy; IL, interleukin; IBD, inflammatory bowel disease.

## Data Availability

Not applicable.

## References

[1] Rosi E., Fastame M.T., Scandagli I., Di Cesare A., Ricceri F., Pimpinelli N., Prignano F. (2021). Insights into the Pathogenesis of HS and Therapeutical Approaches. Biomedicines.

[2] Naik H.B., Nassif A., Ramesh M.S., Schultz G., Piguet V., Alavi A., Lowes M.A. (2019). Are Bacteria Infectious Pathogens in Hidradenitis Suppurativa? Debate at the Symposium for Hidradenitis Suppurativa Advances Meeting, November 2017. J. Invest. Dermatol..

[3] Coates M., Mariottoni P., Corcoran D.L., Kirshner H.F., Jaleel T., Brown D.A., Brooks S.R., Murray J., Morasso M.I., MacLeod A.S. (2019). The Skin Transcriptome in Hidradenitis Suppurativa Uncovers an Antimicrobial and Sweat Gland Gene Signature Which Has Distinct Overlap with Wounded Skin. PLoS ONE.

[4] Shanmugam V.K., Jones D., McNish S., Bendall M.L., Crandall K.A. (2019). Transcriptome Patterns in Hidradenitis Suppurativa: Support for the Role of Antimicrobial Peptides and Interferon Pathways in Disease Pathogenesis. Clin. Exp. Dermatol..

[5] Chopra D., Arens R.A., Amornpairoj W., Lowes M.A., Tomic-Canic M., Strbo N., Lev-Tov H., Pastar I. (2022). Innate Immunity and Microbial Dysbiosis in Hidradenitis Suppurativa—Vicious Cycle of Chronic Inflammation. Front. Immunol..

[6] Williams S.C., Frew J.W., Krueger J.G. (2021). A Systematic Review and Critical Appraisal of Metagenomic and Culture Studies in Hidradenitis Suppurativa. Exp. Dermatol..

[7] Ring H.C., Mikkelsen P.R., Miller I.M., Jenssen H., Fuursted K., Saunte D.M., Jemec G.B.E. (2015). The Bacteriology of Hidradenitis Suppurativa: A Systematic Review. Exp. Dermatol..

[8] Nikolakis G., Join-Lambert O., Karagiannidis I., Guet-Revillet H., Zouboulis C.C., Nassif A. (2015). Bacteriology of Hidradenitis Suppurativa/Acne Inversa: A Review. J. Am. Acad. Dermatol..

[9] Ring H.C., Emtestam L. (2016). The Microbiology of Hidradenitis Suppurativa. Dermatol. Clin..

[10] Wark K.J.L., Cains G.D. (2021). The Microbiome in Hidradenitis Suppurativa: A Review. Dermatol. Ther..

[11] Schell S.L., Schneider A.M., Nelson A.M. (2021). Yin and Yang: A Disrupted Skin Microbiome and an Aberrant Host Immune Response in Hidradenitis Suppurativa. Exp. Dermatol..

[12] Luck M.E., Tao J., Lake E.P. (2022). The Skin and Gut Microbiome in Hidradenitis Suppurativa: Current Understanding and Future Considerations for Research and Treatment. Am. J. Clin. Dermatol..

[13] Mintoff D., Borg I., Pace N.P. (2021). The Clinical Relevance off the Microbiome in Hidradenitis Suppurativa: A Systematic Review. Vaccines.

[14] Highet A.S., Warren R.E., Weekes A.J. (1988). Bacteriology and Antibiotic Treatment of Perineal Suppurative Hidradenitis. Arch. Dermatol..

[15] Jemec G.B., Faber M., Gutschik E., Wendelboe P. (1996). The Bacteriology of Hidradenitis Suppurativa. Dermatology.

[16] Katoulis A., Koumaki V., Efthymiou O., Koumaki D., Dimitroulia E., Voudouri M., Voudouri A., Bozi E., Tsakris A. (2020). Staphylococcus Aureus Carriage Status in Patients with Hidradenitis Suppurativa: An Observational Cohort Study in a Tertiary Referral Hospital in Athens, Greece. Dermatology.

[17] Dinh K.M., Erikstrup L.T., Andersen R.K., Andersen P.S., Mikkelsen S., Kjerulff B.D., Burgdorf K.S., Hansen T.F., Nielsen K.R., Hjalgrim H. (2020). Cross-Sectional Study Identifies Lower Risk of Staphylococcus Aureus Nasal Colonization in Danish Blood Donors with Hidradenitis Suppurativa Symptoms. Br. J. Dermatol..

[18] Stergianou D., Tzanetakou V., Argyropoulou M., Kanni T., Bagos P.G., Giamarellos-Bourboulis E.J. (2021). Staphylococcus Aureus Carriage in Hidradenitis Suppurativa: Impact on Response to Adalimumab. Dermatology.

[19] Hsu T.-J., Yeh H.-H., Lee C.-H., Tseng H.-C. (2022). The Temporal Evolution of Distinct Skin Surface Microbiome in Asian Patients with Severe Hidradenitis Suppurativa during Effective Adalimumab Treatment. J. Invest. Dermatol..

[20] Corazza M., Borghi A., Bettoli V., Pora R., Bononi I., Mazzoni E., Mazzola E., Saraceni S., Maritati M., Contini C. (2021). Irrelevance of Panton-Valentine Leukocidin in Hidradenitis Suppurativa: Results from a Pilot, Observational Study. Eur. J. Clin. Microbiol. Infect. Dis..

[21] Guenin-Macé L., Morel J.-D., Doisne J.-M., Schiavo A., Boulet L., Mayau V., Goncalves P., Duchatelet S., Hovnanian A., Bondet V. (2020). Dysregulation of Tryptophan Catabolism at the Host-Skin Microbiota Interface in Hidradenitis Suppurativa. JCI Insight.

[22] Brook I., Frazier E.H. (1999). Aerobic and Anaerobic Microbiology of Axillary Hidradenitis Suppurativa. J. Med. Microbiol..

[23] Lapins J., Jarstrand C., Emtestam L. (1999). Coagulase-Negative Staphylococci Are the Most Common Bacteria Found in Cultures from the Deep Portions of Hidradenitis Suppurativa Lesions, as Obtained by Carbon Dioxide Laser Surgery. Br. J. Dermatol..

[24] Sartorius K., Killasli H., Oprica C., Sullivan A., Lapins J. (2012). Bacteriology of Hidradenitis Suppurativa Exacerbations and Deep Tissue Cultures Obtained during Carbon Dioxide Laser Treatment. Br. J. Dermatol..

[25] Matusiak Ł., Bieniek A., Szepietowski J.C. (2014). Bacteriology of Hidradenitis Suppurativa—Which Antibiotics Are the Treatment of Choice?. Acta Derm. Venereol..

[26] Katoulis A.C., Koumaki D., Liakou A.I., Vrioni G., Koumaki V., Kontogiorgi D., Tzima K., Tsakris A., Rigopoulos D. (2015). Aerobic and Anaerobic Bacteriology of Hidradenitis Suppurativa: A Study of 22 Cases. Ski. Appendage Disord..

[27] Guet-Revillet H., Coignard-Biehler H., Jais J.-P., Quesne G., Frapy E., Poirée S., Le Guern A.-S., Le Flèche-Matéos A., Hovnanian A., Consigny P.-H. (2014). Bacterial Pathogens Associated with Hidradenitis Suppurativa, France. Emerg. Infect. Dis..

[28] Ardon C.B., Prens E.P., Fuursted K., Ejaz R.N., Shailes J., Jenssen H., Jemec G.B.E. (2019). Biofilm Production and Antibiotic Susceptibility of Staphylococcus Epidermidis Strains from Hidradenitis Suppurativa Lesions. J. Eur. Acad. Dermatol. Venereol..

[29] Ardon C.B., Prens E.P., Tkadlec J., Fuursted K., Abourayale S., Jemec G.B.E., Jenssen H. (2019). Virulent Staphylococcus Lugdunensis with Limited Genetic Diversity in Hidradenitis Suppurativa Lesions. J. Eur. Acad. Dermatol. Venereol..

[30] Nikolakis G., Liakou A.I., Bonovas S., Seltmann H., Bonitsis N., Join-Lambert O., Wild T., Karagiannidis I., Zolke-Fischer S., Langner K. (2017). Bacterial Colonization in Hidradenitis Suppurativa/Acne Inversa: A Cross-Sectional Study of 50 Patients and Review of the Literature. Acta Derm. Venereol..

[31] Benzecry V., Grancini A., Guanziroli E., Nazzaro G., Barbareschi M., Marzano A.V., Muratori S., Veraldi S. (2020). Hidradenitis Suppurativa/Acne Inversa: A Prospective Bacteriological Study and Review of the Literature. G. Ital. Dermatol. Venereol..

[32] Naik H.B., Jo J.-H., Paul M., Kong H.H. (2020). Skin Microbiota Perturbations Are Distinct and Disease Severity-Dependent in Hidradenitis Suppurativa. J. Invest. Dermatol..

[33] Riverain-Gillet É., Guet-Revillet H., Jais J.-P., Ungeheuer M.-N., Duchatelet S., Delage M., Lam T., Hovnanian A., Nassif A., Join-Lambert O. (2020). The Surface Microbiome of Clinically Unaffected Skinfolds in Hidradenitis Suppurativa: A Cross-Sectional Culture-Based and 16S RRNA Gene Amplicon Sequencing Study in 60 Patients. J. Invest. Dermatol..

[34] Hessam S., Sand M., Georgas D., Anders A., Bechara F.G. (2016). Microbial Profile and Antimicrobial Susceptibility of Bacteria Found in Inflammatory Hidradenitis Suppurativa Lesions. Ski. Pharmacol. Physiol..

[35] Mahdi J., Nasrin S., Farnoosh N. (2019). Microbial Profile and Antibiotic Susceptibility of Bacteria Isolated from Patients with Hidradenitis Suppurativa. Iran. J. Dermatol..

[36] Guet-Revillet H., Jais J.-P., Ungeheuer M.-N., Coignard-Biehler H., Duchatelet S., Delage M., Lam T., Hovnanian A., Lortholary O., Nassif X. (2017). The Microbiological Landscape of Anaerobic Infections in Hidradenitis Suppurativa: A Prospective Metagenomic Study. Clin. Infect. Dis..

[37] Ring H.C., Sigsgaard V., Thorsen J., Fuursted K., Fabricius S., Saunte D.M., Jemec G.B. (2019). The Microbiome of Tunnels in Hidradenitis Suppurativa Patients. J. Eur. Acad. Dermatol. Venereol..

[38] Ring H.C., Thorsen J., Saunte D.M., Lilje B., Bay L., Riis P.T., Larsen N., Andersen L.O., Nielsen H.V., Miller I.M. (2017). The Follicular Skin Microbiome in Patients with Hidradenitis Suppurativa and Healthy Controls. JAMA Dermatol..

[39] Schneider A.M., Cook L.C., Zhan X., Banerjee K., Cong Z., Imamura-Kawasawa Y., Gettle S.L., Longenecker A.L., Kirby J.S., Nelson A.M. (2020). Loss of Skin Microbial Diversity and Alteration of Bacterial Metabolic Function in Hidradenitis Suppurativa. J. Invest. Dermatol..

[40] Antal D., Janka E.A., Szabó J., Szabó I.L., Szegedi A., Gáspár K., Bai P., Szántó M. (2022). Culture-Based Analyses of Skin Bacteria in Lesional Moist, and Unaffected Dry and Sebaceous Skin Regions of Hidradenitis Suppurativa Patients. J. Eur. Acad. Dermatol. Venereol..

[41] Bettoli V., Manfredini M., Massoli L., Carillo C., Barozzi A., Amendolagine G., Ruina G., Musmeci D., Libanore M., Curtolo A. (2019). Rates of Antibiotic Resistance/Sensitivity in Bacterial Cultures of Hidradenitis Suppurativa Patients. J. Eur. Acad. Dermatol. Venereol..

[42] Jahns A.C., Killasli H., Nosek D., Lundskog B., Lenngren A., Muratova Z., Emtestam L., Alexeyev O.A. (2014). Microbiology of Hidradenitis Suppurativa (Acne Inversa): A Histological Study of 27 Patients. APMIS.

[43] Okoye G.A., Vlassova N., Olowoyeye O., Agostinho A., James G., Stewart P.S., Leung S., Lazarus G. (2017). Bacterial Biofilm in Acute Lesions of Hidradenitis Suppurativa. Br. J. Dermatol..

[44] Ring H.C., Bay L., Nilsson M., Kallenbach K., Miller I.M., Saunte D.M., Bjarnsholt T., Tolker-Nielsen T., Jemec G.B. (2017). Bacterial Biofilm in Chronic Lesions of Hidradenitis Suppurativa. Br. J. Dermatol..

[45] Ring H.C., Bay L., Kallenbach K., Miller I.M., Prens E., Saunte D.M., Bjarnsholt T., Jemec G.B.E. (2017). Normal Skin Microbiota Is Altered in Pre-Clinical Hidradenitis Suppurativa. Acta Derm. Venereol..

[46] Kjaersgaard Andersen R., Ring H.C., Kallenbach K., Eriksen J.O., Jemec G.B.E. (2019). Bacterial Biofilm Is Associated with Higher Levels of Regulatory T Cells in Unaffected Hidradenitis Suppurativa Skin. Exp. Dermatol..

[47] Ring H.C., Thorsen J., Saunte D.M., Lilje B., Bay L., Riis P.T., Larsen N., Andersen L.O., Nielsen H.V., Miller I.M. (2018). Moderate to Severe Hidradenitis Suppurativa Patients Do Not Have an Altered Bacterial Composition in Peripheral Blood Compared to Healthy Controls. J. Eur. Acad. Dermatol. Venereol..

[48] Hispán P., Murcia O., Gonzalez-Villanueva I., Francés R., Giménez P., Riquelme J., Betlloch I., Pascual J.C. (2020). Identification of Bacterial DNA in the Peripheral Blood of Patients with Active Hidradenitis Suppurativa. Arch. Dermatol. Res..

[49] Kam S., Collard M., Lam J., Alani R.M. (2021). Gut Microbiome Perturbations in Patients with Hidradenitis Suppurativa: A Case Series. J. Invest. Dermatol..

[50] Eppinga H., Weiland C.J.S., Thio H.B., van der Woude C.J., Nijsten T.E.C., Peppelenbosch M.P., Konstantinov S.R. (2016). Similar Depletion of Protective Faecalibacterium Prausnitzii in Psoriasis and Inflammatory Bowel Disease, but Not in Hidradenitis Suppurativa. J. Crohns Colitis.

[51] Lam S.Y., Radjabzadeh D., Eppinga H., Nossent Y.R.A., van der Zee H.H., Kraaij R., Konstantinov S.R., Fuhler G.M., Prens E.P., Thio H.B. (2021). A Microbiome Study to Explore the Gut-Skin Axis in Hidradenitis Suppurativa. J. Dermatol. Sci..

[52] McCarthy S., Barrett M., Kirthi S., Pellanda P., Vlckova K., Tobin A.-M., Murphy M., Shanahan F., O’Toole P.W. (2022). Altered Skin and Gut Microbiome in Hidradenitis Suppurativa. J. Invest. Dermatol..

[53] Giamarellos-Bourboulis E.J. (2019). Staphylococcus Aureus and Host Interaction in the Flare-Ups of Hidradenitis Suppurativa. Br. J. Dermatol..

[54] Ring H.C., Thorsen J., Fuursted K., Bjarnsholt T., Bay L., Egeberg A., Ingham A.C., Vedel Nielsen H., Frew J.W., Saunte D.M.L. (2022). Amplicon Sequencing Demonstrates Comparable Follicular Mycobiomes in Patients with Hidradenitis Suppurativa Compared with Healthy Controls. J. Eur. Acad. Dermatol. Venereol..

[55] Jørgensen A.-H.R., Thomsen S.F., Karmisholt K.E., Ring H.C. (2020). Clinical, Microbiological, Immunological and Imaging Characteristics of Tunnels and Fistulas in Hidradenitis Suppurativa and Crohn’s Disease. Exp. Dermatol..

[56] Ring H.C., Thorsen J., Jørgensen A.H., Bay L., Bjarnsholt T., Fuursted K., Thomsen S.F., Jemec G.B. (2020). Predictive Metagenomic Analysis Reveals a Role of Cutaneous Dysbiosis in the Development of Hidradenitis Suppurativa. J. Invest. Dermatol..

[57] Schneider A.M., Cook L.C., Zhan X., Banerjee K., Cong Z., Imamura-Kawasawa Y., Gettle S.L., Longenecker A.L., Kirby J.S., Nelson A.M. (2020). Response to Ring et al.: In Silico Predictive Metagenomic Analyses Highlight Key Metabolic Pathways Impacted in the Hidradenitis Suppurativa Skin Microbiome. J. Invest. Dermatol..

[58] Ring H.C., Thorsen J., Fuursted K., Bjarnsholt T., Bay L., Saunte D.M., Thomsen S.F., Jemec G.B. (2022). Probiotics in Hidradenitis Suppurativa: A Potential Treatment Option?. Clin. Exp. Dermatol..

[59] Maraki S., Evangelou G., Stafylaki D., Scoulica E. (2017). Actinotignum Schaalii Subcutaneous Abscesses in a Patient with Hidradenitis Suppurativa: Case Report and Literature Review. Anaerobe.

[60] Bonifaz A., Fernández-Samar D., Tirado-Sánchez A., Vázquez-González D., Mercadillo-Pérez P. (2021). Hidradenitis Suppurativa Associated with Actinomycosis Owing to Actinomyces Meyeri. Br. J. Dermatol..

[61] Kathju S., Lasko L.-A., Stoodley P. (2012). Considering Hidradenitis Suppurativa as a Bacterial Biofilm Disease. FEMS Immunol. Med. Microbiol..

[62] Iwase T., Uehara Y., Shinji H., Tajima A., Seo H., Takada K., Agata T., Mizunoe Y. (2010). Staphylococcus Epidermidis Esp Inhibits Staphylococcus Aureus Biofilm Formation and Nasal Colonization. Nature.

[63] Molnar J., Mallonee C.J., Stanisic D., Homme R.P., George A.K., Singh M., Tyagi S.C. (2020). Hidradenitis Suppurativa and 1-Carbon Metabolism: Role of Gut Microbiome, Matrix Metalloproteinases, and Hyperhomocysteinemia. Front. Immunol..

[64] Naik H.B., Piguet V. (2020). Standardizing Hidradenitis Suppurativa Skin Microbiome Research: The Methods Matter. J. Invest. Dermatol..

[65] Langan E.A., Recke A., Bokor-Billmann T., Billmann F., Kahle B.K., Zillikens D. (2020). The Role of the Cutaneous Microbiome in Hidradenitis Suppurativa-Light at the End of the Microbiological Tunnel. Int. J. Mol. Sci..

[66] Niemeyer-van der Kolk T., van der Wall H.E.C., Balmforth C., Van Doorn M.B.A., Rissmann R. (2018). A Systematic Literature Review of the Human Skin Microbiome as Biomarker for Dermatological Drug Development. Br. J. Clin. Pharmacol..

[67] Delage M., Guet-Revillet H., Duchatelet S., Hovnanian A., Nassif X., Nassif A., Join-Lambert O. (2015). Deciphering the Microbiology of Hidradenitis Suppurativa: A Step Forward towards Understanding an Enigmatic Inflammatory Skin Disease. Exp. Dermatol..

[68] Callewaert C., Knödlseder N., Karoglan A., Güell M., Paetzold B. (2021). Skin Microbiome Transplantation and Manipulation: Current State of the Art. Comput. Struct. Biotechnol. J..

